# Stage-Dependent Within-Individual Comparison Reveals SIV-Specific Activation/Exhaustion Shift in Rhesus Macaques

**DOI:** 10.3389/fmicb.2021.704449

**Published:** 2021-07-19

**Authors:** Ling Tong, Zhe Cong, Long Tian, Jingjing Zhang, Jiahan Lu, Qiuhan Lu, Ting Chen, Yuhong Wang, Qiang Wei, Jing Xue

**Affiliations:** ^1^Key Laboratory of Human Disease Comparative Medicine, Chinese Ministry of Health, Beijing Key Laboratory for Animal Models of Emerging and Remerging Infectious Diseases, Institute of Laboratory Animal Science, Chinese Academy of Medical Sciences and Comparative Medicine Center, Peking Union Medical College, Beijing, China; ^2^Department of Gerontology and Geriatrics, The First Affiliated Hospital of Harbin Medical University, Harbin, China; ^3^Center for AIDS Research, Chinese Academy of Medical Sciences, Beijing, China

**Keywords:** HIV/SIV, rhesus macaques, activation, exhaustion, stage-dependent comparison

## Abstract

It is challenging to trace the complicated individual-based variations of HIV-specific immunocompetence shift during the successful antiretroviral therapy (ART) era. Using eight rhesus monkeys simulating a longitudinal stage-dependent cohort (baseline-SIV acute infection-SIV suppression by ART-ART withdrawal), baseline immunocompetence monitoring for 28 days (SIV-negative stage, SN) was compared with host immunocompetence undergoing 90-day ART treatment (SIV-suppressed stage, SS) to reveal the SIV-specific immunity shift aroused by undetectable individual viral replication. During acute SIV infection for 98 days (SIV-emerged stage, SE), immune activation was compared with re-immune activation post ART for 49-day follow-up (SIV-rebounded stage, SR) to reveal the SIV-specific immune activation variation aroused by detectable individual viral replication. Individual immunocompetence was measured by co-expression of CD4, CD8, CD38, HLA-DR, CCR7, CD45RA, and PD-1 on T cells and a cytokine panel. Compared with SN, mild immune activation/exhaustion was characterized by increased CD38^+^ HLA-DR^–^ CD4^+^/CD8^+^ T-cell subsets and PD-1^+^ memory CD4^+^/CD8^+^ T-cell subsets with three elevated cytokines (MIP-1β, IL-8, and IL-10) significantly emerged in SS. Compared with SE, SR produced more exhaustion characterized by increased PD-1^+^ CD4^+^ T_CM_ cells and decreased PD-1^+^ CD4^+^ T_EM_ cells with four elevated pro-inflammatory cytokines (IFN-γ, IL-1β, IL-6, and TNF-α). By such individualized stage-dependent comparison, the sustainable immune activation was found from activation/exhaustion shifted into exhaustion during the longitudinal viral persistence. Further, validated SIV accelerates host immunosenescence continuously independent of viral replication.

## Introduction

As one of the most significant public problems, human immunodeficiency virus type-1 (HIV-1) infection is now associated with long-term subclinical survival due to successful antiretroviral therapy (ART). Acute HIV infection activates CD8^+^ T cells to produce high level systematic inflammation against viral expansion (Douek, 2013; Paiardini and Müller-Trutwin, 2013). Simultaneously, such inflammatory cytokines accelerate CD4^+^ T-cell apoptosis and disturb the immunocompetence balance to progress to immunity deficiency. Despite that, successful ART could draw the host from acute HIV-specific CD8^+^ T-cell activation cascade into an undetectable antigenic presentation and very low response, the “invisible” latency sustainably arouses immunologic activation/exhaustion of memory T cells, the process of which is similar to normal aging (Appay et al., 2007; Pathai et al., 2014). Three attenuated mechanisms on antigen-specific immunity competence, including clonal deletion, functional unresponsiveness (exhaustion), and viral persistence with evaded recognition, were simultaneously studied on chronic ART-treated HIV patients (Goepfert et al., 2000; Goulder et al., 2000; Kostense et al., 2001, 2002). Among them, exhaustion remarkedly showed overexpressed checkpoint makers, particularly programmed death 1 (PD-1) (Brenchley et al., 2006). Elevated PD-1 expression on HIV-specific CD8^+^ T cells plays the role of exhaustion mediator of CD8^+^ T cells to reduce cytokine production and proliferation (Kaufmann and Walker, 2008). Interestingly, PD-1 upregulation linearly correlates with HIV-specific memory CD8^+^ T-cell exhaustion in the acute infection stage but not in long-term non-progressors (Zhang et al., 2007), indicating that chronic virus–host molecular interaction could be more complicated and subtle corresponding to viral persistence. Increasing proofs indicate that continuous high expression of immunoregulatory markers, including PD-1 and other checkpoint receptors, supports sustainable activation/exhaustion during chronic viral persistence (Day et al., 2006; Kaufmann and Walker, 2008).

However, in the context of immunocompetence imbalance, it has not been determined how exhausted T cells end to apoptosis or regain mild activation (Zhang et al., 2007; Youngblood et al., 2013; Hoffmann et al., 2016; Muenchhoff et al., 2019). These differentiation-independent activation/exhaustion/apoptosis are of high between-host heterogeneity (Zhang et al., 2007; Petrovas et al., 2009; Youngblood et al., 2013; Muenchhoff et al., 2019). Moreover, HIV-specific immune activation and CD8^+^ T cytotoxic response in HIV-undetectable status are different between ART-treated individuals and “primed” elite controllers (Sáez-Cirión and Sereti, 2021). As antigen presentation provides costimulatory signals to maintain CD8^+^ T-cell polyfunctionality to resist apoptosis, ART treatment reduces polyfunctionality due to decreased antigenic stimulation instead of stabilization on T-cell perturbation. These CD8^+^ T cells present a low cytotoxic response against HIV and high susceptibility to apoptosis, as apoptosis-resistant CD8^+^ T cells isolated from elite controllers are primed to control HIV replication (Lichterfeld et al., 2007; Streeck et al., 2009). Therefore, the individual-based host–virus interaction shift cannot be achieved by comparing ART-treated HIV carriers and HIV-controlled elite patients due to “primed” genetic variation. The best strategy to reveal such eventual immunocompetence shift from dominant immune activation into sustainable activation/exhaustion until exhaustion/senescence during HIV infection is to monitor the baseline immunity before individual infection. Moreover, longitudinal studies show the global shift from robustness into exhaustion due to attenuated antigen presentation binding costimulatory signals (van der Most et al., 2003). However, such chronic exhaustion does not equate to irreversible termination or deletion. Some exhausted CD8^+^ T cells could circulate long in the peripheral blood with compromised yet sustained polyfunctionality, highly similar to immunosenescent phenotypes (Cao et al., 2016; Martínez-Zamudio et al., 2021).

HIV latency-dependent persistence promotes similar T-cell phenotype shift to normal aging (Deeks, 2011) as chronic low-level inflammatory activation also termed as “inflamm-aging” (Monti et al., 2017). One of the attributive pathogens is cytomegalovirus (CMV), considered responsible for the T-cell immune activation and exhaustion by aging in the general population. Notably, subclinical HIV carriers are more susceptible to age-related cardiovascular disease, metabolic disorders, neurocognitive decline, and cancers due to T-cell immunosenescence (Hansson, 2005; Deeks et al., 2013; van den Dries et al., 2017). From this aspect, HIV persistence is regarded as accelerated immunosenescence (Appay et al., 2007; Molina-Pinelo et al., 2009; Dock and Effros, 2011; Pathai et al., 2014). A bundle of proofs indicates that old asymptomatic HIV carriers who have undergone successful long-term ART harbor heavier loads of aging-related diseases compared with their age-gender matches (Schouten et al., 2014; Appay and Sauce, 2017). Notably, CMV, regarded as the responsible antigen for “natural” immunity aging, does not aggravatedly compromise the CD8^+^ T-cell response as HIV does within the same host (Naeger et al., 2010). However, chronic CMV persistence contributes to more accumulation of immunosenescence and inflammation than HIV does within the same host (Booiman et al., 2017). Such CMV-HIV co-persistence further aggravates the complexity of chronic HIV immunity reconstitution. Within-individual comparison is required to find the optimal balance between antigen-dependent costimulatory signals and effective cytokine signals during an individualized scenario.

Using our individual-based comparison, we attempt to differentiate the overlapped process between T-cell exhaustion and activation and reveal the potentially optimal balance of activation/exhaustion/senescence along with the individual scenario. In the present study, we overtook baseline profiling to “replace” conventional elite controllers, which could adjust residual immunocompetence to achieve within-individual differences before and after ART. We attempt to reveal the immunocompetence shift from host–virus interaction. Also, we compared differences between acute infection and ART-ceased rebound to replenish the “additional” shift of the imbalance of immunity activation/exhaustion/senescence during SIV infection.

## Materials and Methods

### Study Design

Eight 4--6-year-old, male and female pathogen-free (SPF) rhesus monkeys (*Macaca mulatta*) were housed and cared for following the Institutional Animal Care and Use Committee (IACUC) of the Institute of Laboratory Animal Science and the recommendations of the Weatherall report for the use of non-human primates in research^1^ at the Association for Assessment and Accreditation of Laboratory Animal Care (AAALAC)-accredited facility. Macaques were intravenously infected with 100 tissue culture infective doses (TCID_50_) of SIVmac239 as described (Chong et al., 2019). All animal procedures and experiments were performed following protocols approved by the IACUC of the Institute of Laboratory Animal Science, Chinese Academy of Medical Sciences (No. XJ19005). All animals were anesthetized with ketamine hydrochloride (10 mg/kg) before sample collection, and experiments were performed in a biosafety level 3 laboratory.

**FIGURE 1:**
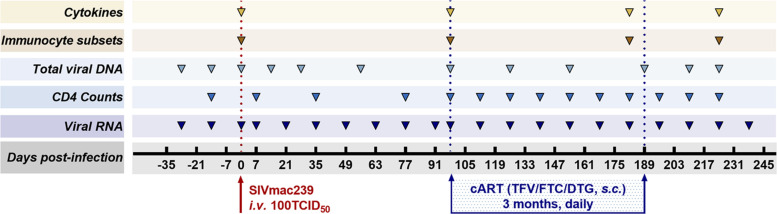
Study design. Eight Chinese-origin rhesus macaques were intravenously (*i.v.*) inoculated with 100 median tissue culture infectious doses (TCID_50_) of SIVmac239. At 98 days post-infection (dpi), antiretroviral therapy (ART) (FTC/DTG/TDF) was administered for 3 months. ART was discontinued at 189 dpi with a 49-day follow-up. Peripheral blood was collected to measure viral RNA, CD4 T-cell counts, total viral DNA, immunocyte subsets, and cytokines at the indicated time points during the observation period.

Antiretroviral therapy was initiated 98 days after infection and continued for 3 months. The ART regimen consisting of two reverse transcriptase inhibitors, 5 mg/ml tenofovir disoproxil fumarate (TDF) and 40 mg/ml emtricitabine (FTC), plus 2.5 mg/ml of the integrase inhibitor dolutegravir (DTG), was subcutaneously administered once daily at 1 ml/kg body weight (Whitney et al., 2018). The eight monkeys were followed up 49 days after discontinuation of ART. As part of the longitudinal observation, the effect of SIV RNA and total viral DNA on CD4 T-cell counts, immunocyte subsets, and cytokines was measured at the indicated time points (Figure 1).

### Quantification of SIV RNA and Total SIV DNA

Viral RNA (vRNA) was isolated from plasma using a QIAamp viral RNA mini kit (Qiagen, Valencia, CA, United States). Total viral DNA (vDNA) was extracted from monkey peripheral blood mononuclear cells (PBMCs) using a QIAamp Blood DNA mini kit (Qiagen, Valencia, CA, United States) as previously reported (Chong et al., 2019). Viral RNA was subjected to quantitative real-time reverse transcription-PCR (qRT-PCR) on an ABI 9700 real-time PCR system (Applied Biosystems) using the following primers and probe: Gag91 forward primer: 5′–GCA GAG GAG GAA ATT ACC CAG TAC–3′; Gag91 reverse primer: 5′–CAA TTT TAC CCA GGC ATT TAA TGT T–3′; Probe: 5′-(FAM)-ACC TGC CAT TAA GCC CGA—(MGB)-3′. The copy numbers were estimated by comparison to a pGEM-SIV gag477 standard curve. The limits of detection were 100 copy equivalents of RNA or DNA per ml of plasma. Triplicate test reactions were performed for each sample.

### Flow Cytometry

**FIGURE 2:**
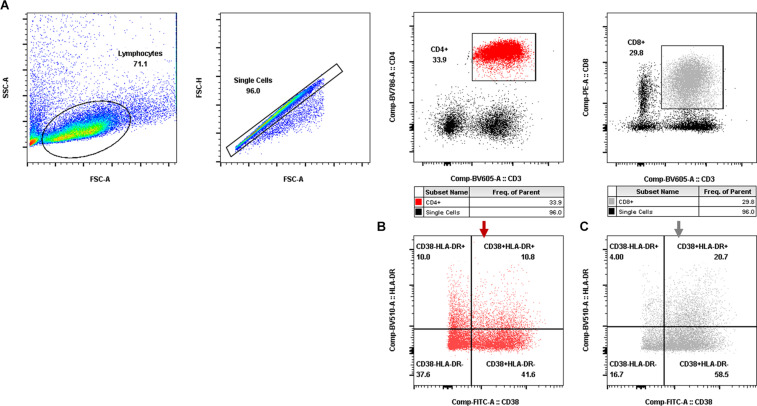
Gating for activated T-cell populations by flow cytometry. **(A)** Gating for CD4^+^ T cells (red dot plot in the rectangle) and CD8^+^ T cells (gray dot plot in the rectangle). **(B,C)** Gating for activated CD4^+^ T-cell subsets **(B)** and activated CD8^+^ T-cell subsets **(C)** with CD38 and/or HLA-DR expression.

**FIGURE 3:**
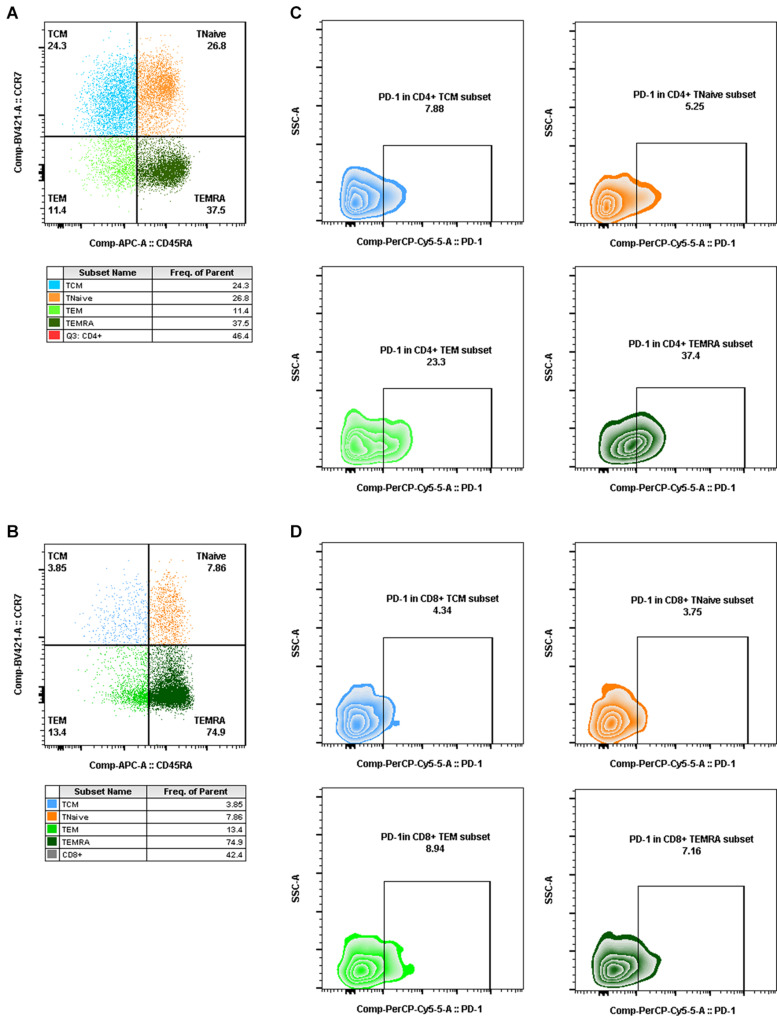
Gating for memory T-cell subsets and PD-1^+^ cells in memory T-cell subsets by flow cytometry. **(A,B)** Gating for CD4^+^ memory T-cell subsets **(A)** and CD8^+^ memory T-cell subsets **(B)** with CCR7 and CD45RA expression (T_CM_, CD4^+^/CD8^+^ CCR7^+^CD45RA^–^, blue dot plot; T_Na__ï__ve_, CD4^+^/CD8^+^ CCR7^+^CD45RA^+^, orange dot plot; T_EM_, CD4^+^/CD8^+^ CCR7^–^CD45RA^–^, light green dot plot; T_EMRA_, CD4^+^/CD8^+^ CCR7^–^CD45RA^+^, dark green dot plot). **(C,D)** Gating for PD-1^+^ cells in CD4^+^ memory T-cell subsets **(C)** and PD-1^+^ cells in CD8^+^ memory T-cell subsets **(D)** (PD-1^+^ T_CM_, CD4^+^/CD8^+^ PD-1^+^CCR7^+^CD45RA^–^, blue zebra plot; PD-1^+^ T_Na__ï__ve_, CD4^+^/CD8^+^ PD-1^+^ CCR7^+^CD45RA^+^, orange zebra plot; PD-1^+^ T_EM_, CD4^+^/CD8^+^ PD-1^+^ CCR7^–^CD45RA^–^, light green zebra plot; PD-1^+^ T_EMRA_, CD4^+^/CD8^+^ PD-1^+^ CCR7^–^CD45RA^+^, dark green zebra plot).

Aliquots (50 μl) of EDTA-treated whole blood were stained with monoclonal antibodies to CD3 PerCP (SP34-2, BD Biosciences, 552851), CD4 FITC (OKT-4, Biolegend, 317408), and CD8 PE (RPA-T8, BD Biosciences, 555367). CD4^+^ T-cell counts were determined with BD Trucount tubes according to the manufacturer’s instructions (BD Biosciences, San Diego, CA, United States). PBMCs were isolated using conventional Ficoll-Hypaque density gradient centrifugation (GE Healthcare, Uppsala, Sweden). Polychromatic flow cytometry was performed to quantitate activated CD4^+^ or CD8^+^ T lymphocytes (Figure 2) and CD4^+^ or CD8^+^ memory T lymphocyte subsets (Figure 3). Activated or memory T lymphocyte subsets (Table 1) from 1 × 10^6^ PBMCs were stained with anti-CD3 BV605 (SP34-2, BD Biosciences, 562994), anti-CD4 BV711 (OKT-4, Biolegend, 317440), anti-CD8 PE (RPA-T8, BD Biosciences, 557086), anti-CCR7 BV421 (G043H7, Biolegend, 352208), anti-CD45RA APC (5H9, BD Biosciences, 561210), anti-CD38 FITC (AT-1, Stemcell, 60131FI), anti-HLA-DR BV510 (G46-6, BD Biosciences, 563083), and anti-PD-1 PerCP-cy5.5 (EH12.2H7, Biolegend, 329914) monoclonal antibodies. Cells were resuspended in 1% paraformaldehyde, subjected to flow cytometry within 24 h on a FACSAriaII (BD Biosciences, San Diego, CA, United States), and analyzed using FlowJo V_10_ software.

**TABLE 1:** Polychromatic flow cytometry for staining of T lymphocyte subsets.

T lymphocyte subsets	Biomarker
Activated T cells	CD4^+^/CD8^+^ CD38^+^ HLA-DR^+^
	CD4^+^/CD8^+^ CD38^+^ HLA-DR^–^
	CD4^+^/CD8^+^ CD38^–^ HLA-DR^+^
	CD4^+^/CD8^+^ CD38^–^ HLA-DR^–^
Naive T cells (T_Naive_)	CD3^+^ CD4^+^/CD8^+^ CCR7^+^ CD45RA^+^
PD-1^+^ T_Naive_	CD3^+^ CD4^+^/CD8^+^ CCR7^+^ CD45RA^+^ PD-1^+^
Central memory T cells (T_CM_)	CD3^+^ CD4^+^/CD8^+^ CCR7^+^ CD45RA^–^
PD-1^+^ T_CM_	CD3^+^ CD4^+^/CD8^+^ CCR7^+^ CD45RA^–^ PD-1^+^
Effector memory RA^+^ T cells (T_EMRA_)	CD3^+^ CD4^+^/CD8^+^ CCR7^–^ CD45RA^+^
PD-1^+^ T_EMRA_	CD3^+^ CD4^+^/CD8^+^ CCR7^–^ CD45RA^+^ PD-1^+^
Effective memory T cells (T_EM_)	CD3^+^ CD4^+^/CD8^+^ CCR7^–^ CD45RA^–^
PD-1^+^ T_EM_	CD3^+^ CD4^+^/CD8^+^ CCR7^–^ CD45RA^–^ PD-1^+^

### Multiplex Analysis Using Luminex

Blood samples were centrifuged for 10 min at 600 × *g*, and serum was immediately aliquoted and stored at −80°C. The following 11 cytokines were measured with a Luminex kit following the manufacturer’s instructions: IL-1β, IL-2, IL-6, IL-8, IL-10, IFN-γ, MCP-1, MIP-1β, TNF-α (Merck Millipore, Billerica, MA, United States, PRCYTOMAG-40K-09C), TGF-β (Merck Millipore, Billerica, MA, United States, TGFBMAG-64K-01), and IP-10 (Carlsbad, CA, United States, EPX01A-40284-901). After thawing the samples on ice and sufficient mixing, 25 μl of supernatant was loaded into each well of a 96-well plate and mixed with 25 μl of assay buffer and 25 μl of magnetic beads. The plates were incubated with agitation overnight at 4°C. After washing, 25 μl of detection antibody was added to each well, and the plate was incubated for 1 h at room temperature (RT). Then, 25 μl of streptavidin-PE was added to each well and incubated for 30 min at RT. Next, 150 μl sheath fluid was added to each well after washing. Plates were read on a Luminex^®^ 200 (Bio-Rad, Hercules, CA, United States), and the data were analyzed for median fluorescent intensity using a five-parameter logistic method for calculating analyte concentration.

### Statistical Analysis

Comparisons between the two groups were determined using paired *t*-tests. Comparison of quantitative variables was assessed with Friedman’s test. The Spearman rank test was used to determine correlations. All data were analyzed using GraphPad Prism 9.0 software (GraphPad Software Inc., San Diego, CA, United States). Significance was set at ^∗^*P* < 0.05, ^∗∗^*P* < 0.01, and ^∗∗∗^*P* < 0.001.

## Results

### Generation of the ART-Treated, SIVmac239-Infected Monkey Model

**FIGURE 4:**
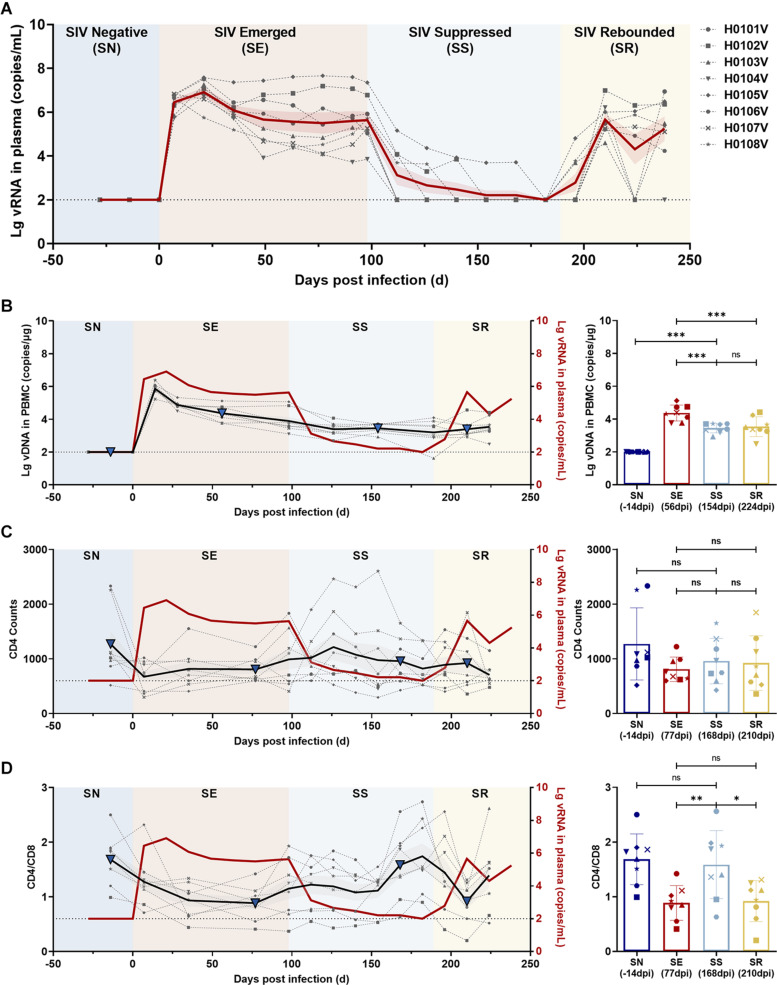
Dynamics of viral RNA, viral DNA, CD4 T-cell counts, and CD4/CD8 ratio in ART-treated, SIVmac239-infected macaques. **(A)** Dynamics of viral RNA replication. The observation period was divided into four phases according to viral replication: SIV negative (SN), SIV emerged (SE), SIV suppressed (SS), and SIV rebounded (SR). The gray dotted lines indicate the changes in viral RNA in each monkey; the solid red line, the average of viral loads for all monkeys; the red shaded area, the SEM of viral loads for all monkeys. **(B–D)** Dynamics of total viral DNA, CD4 T-cell counts, and CD4/CD8 ratio during the observation period. Left panel, the changes in **(B)** total viral DNA, **(C)** CD4 T-cell counts, and **(D)** CD4/CD8 ratio during the four phases (gray dotted line, changes in each monkey; black solid line, the average of all monkeys; gray shaded areas, the SD of all monkeys; solid red line, the average viral loads of all monkeys). Right panel, comparisons among SN, SE, SS, and SR. The blue triangles in the left panel represent the time points in the right panel. (*P < 0.05, **P < 0.01, ***P < 0.001, paired *t*-test).

Eight rhesus monkeys were intravenously infected with 100 TCID_50_ SIVmac239. A 3-month ART regimen consisting of daily *s.c.* injections of TDF, DTG, and FTC was continued from 98 days post-infection (dpi), with a 49-day follow-up after stopping ART. During the observation periods, the levels of viral RNA and total viral DNA were measured, together with counts of CD4^+^ T cells and ratios of immunocyte subsets and quantification of cytokine levels (Figure 1). Data collection for the model was divided into four stages: baseline (pre-infection), infection, treatment, and withdrawal based on the variation of vRNA replication (Figure 4A). Regarding the virus, at the baseline stage, SIV was negative (SN, “baseline”); after acute and chronic SIV infection, SIV emerged (SE, “infection”) with a peak plasma SIV RNA level of 6.91 log_10_ copies/ml (range 5.75–7.58 log_10_ RNA copies/ml); during ART, the SIV replication was suppressed (SS, “treatment”) to undetectable levels (limits of detection, 2.00 log_10_ RNA copies/ml); after cessation of ART, the SIV RNA rebounded (SR, “withdrawal”) with the peak of plasma viral RNA ranging from 4.60 to 6.99 log_10_ RNA copies/ml. The longitudinal vDNA ranged from 3.20 to 3.90 log_10_ vDNA copies/ml after acute SIV infection. The vDNA in the SR phase was significantly lower than in the SE phase (SR vs. SE, *P* = 0.0009) (Figure 4B, *right panel*).

The CD4^+^ T-cell counts and CD4/CD8 ratios were compared to determine the status of immune reconstitution. CD4^+^ T-cell counts during ART (SS phase) were slightly increased, but differences were not significant (SS vs. SE, *P* = 0.4044; and SS vs. SR, *P* = 0.8061) (Figure 4C). CD4/CD8 ratios in the SS phase were significantly increased compared with those during SE (SS vs. SE, *P* = 0.0017) and in the SR phase (SS vs. SR, *P* = 0.0128), which returned to the normal level before infection (SS vs. SN, *P* = 0.4280) (Figure 4D).

### Significant Increase in Activated or PD-1-Expressing T Cells With Elevated Levels of Inflammatory Cytokines in SS vs. SN

**FIGURE 5:**
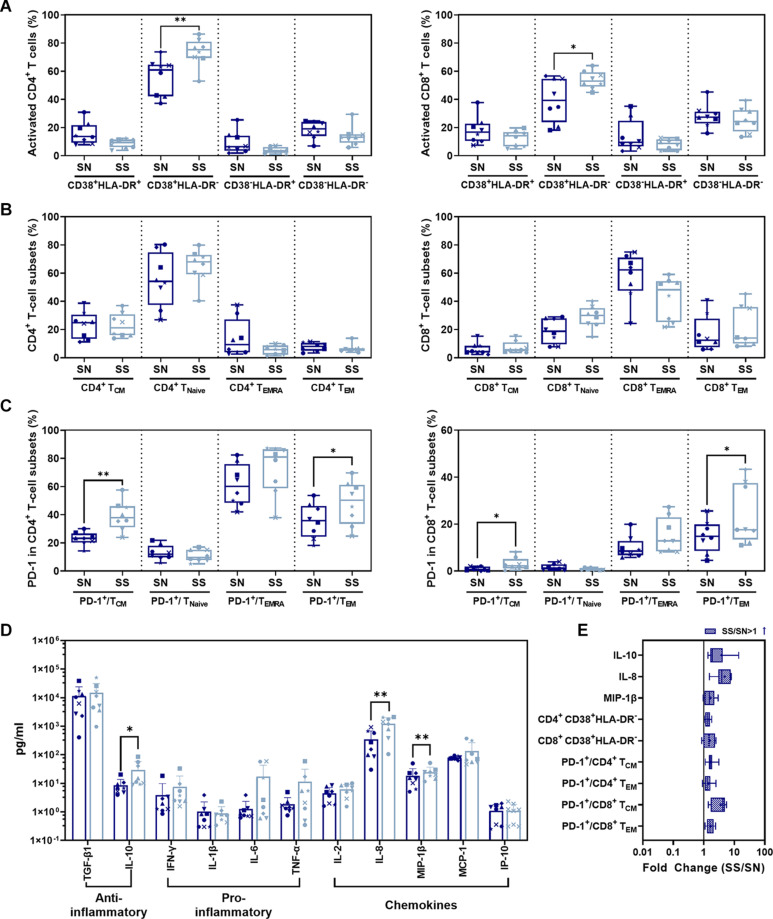
Comparison of CD4^+^/CD8^+^ T-cell subsets and cytokines between SS and SN phases. **(A)** Comparison of activated CD4^+^ (*left panel*) and CD8^+^ (*right panel*) T-cell subsets from PBMC in SS vs. SN. **(B)** Comparison of CD4^+^ (*left panel*) and CD8^+^ (*right panel*) memory T-cell subsets from PBMC in SS vs. SN. The percentage of central memory CD4^+^/CD8^+^ T cells (CD4^+^/CD8^+^ T_CM_, CD3^+^ CD4^+^/CD8^+^ CCR7^+^ CD45RA^–^), naive CD4^+^/CD8^+^ T cells (CD4^+^/CD8^+^ T_Naive_, CD3^+^ CD4^+^/CD8^+^ CCR7^+^ CD45RA^+^), TEMRA cells (CD4^+^/CD8^+^ T_EMRA_, CD3^+^ CD4^+^/CD8^+^ CCR7^–^ CD45RA^+^), and effective memory T cells (CD4^+^/CD8^+^ T_EM_, CD3^+^ CD4^+^/CD8^+^ CCR7^–^ CD45RA^–^) were compared between SS and SN phases (paired *t*-test, *P* > 0.05). **(C)** Comparison of percentages of PD-1^+^ cells in memory T-cell subsets in SS vs. SN. Left panel, comparison of percentages of PD-1^+^ cells in CD4^+^ T_CM_, T_Naive_, T_EMRA_, and T_EM_ cells; right panel, comparison of percentages of PD-1^+^ cells in CD8^+^ T_CM_, T_Naive_, T_EMRA_, and T_EM_ cells. **(D)** Comparison of anti- and pro-inflammatory cytokines and chemokines between SS and SN phases. **(E)** Fold change of cytokines and T-cell subsets with significant differences (SS value/SN value; blue filled patterns represent increased values). Data are the mean ± SD from three independent experiments (^∗^*P* < 0.05, ^∗∗^*P* < 0.01, paired *t*-test, SS vs. SN).

**FIGURE 6:**
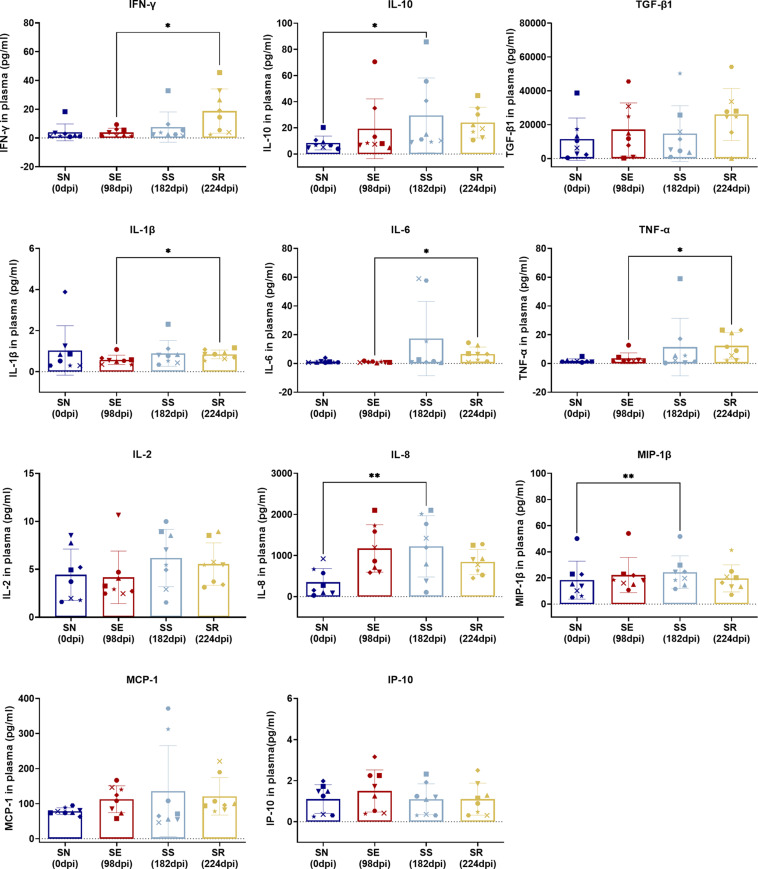
Comparison of cytokines between SS and SN and between SR and SE. Cytokines were detected in the serum of SIV-infected macaques at the indicated phase. Levels (pg/ml) of IFN-γ, IL-10, TGF-β1, IL-1β, IL-6, TNF-α, IL-2, IL-8, MIP-1β, MCP-1, and IP-10 were measured and assessed by paired *t*-test in SS vs. SN and SR vs. SE. Data shown are the mean ± SD from three independent experiments (^∗^*P* < 0.05, ^∗∗^*P* < 0.01).

Ratios of CD38^+^ HLA-DR^–^ CD4^+^ (*P* = 0.0027)/CD8^+^ (*P* = 0.0373) T-cell subsets in the SS phase were significantly higher than in SN before infection (Figures 5A,E). No significant difference was shown in CD38^+^ HLA-DR^+^, CD38^–^ HLA-DR^+^, or CD38^–^ HLA-DR^–^ activated CD4^+^/CD8^+^ T-cell subsets between SS and SN phases (*P* > 0.05). For memory CD4^+^/CD8^+^ T-cell subsets, CD4^+^/CD8^+^ T_CM_, T_N__aive_, T_EMRA_, or T_EM_ during ART (SS) were not significantly different compared to those in SN (Figure 5B). There was a significant increase in PD-1-expressing T-cell subsets during SS including PD-1^+^/CD4^+^ T_CM_ (*P* = 0.0018) and PD-1^+^/CD4^+^ T_EM_ (*P* = 0.0165) (Figure 5C, *left panel* and Figure 5E) as well as PD-1^+^/CD8^+^ T_CM_ (*P* = 0.0270) and PD-1^+^/CD8^+^ T_EM_ (*P* = 0.0106) (Figure 5C, *right panel* and Figure 5E) compared to before infection. Serum profiles of anti- and pro-inflammatory cytokines and chemokines were compared between SS and SN (Figure 6). IL-10 (*P* = 0.0482), IL-8 (*P* = 0.0040), and MIP-1β (*P* = 0.0080) were significantly elevated in SS compared to SN phase (Figures 5D,E).

### Significant Shift in PD-1-Activated T Cells With Elevated Inflammatory Cytokines in Relation to SIV Replication in SR vs. SE

**FIGURE 7:**
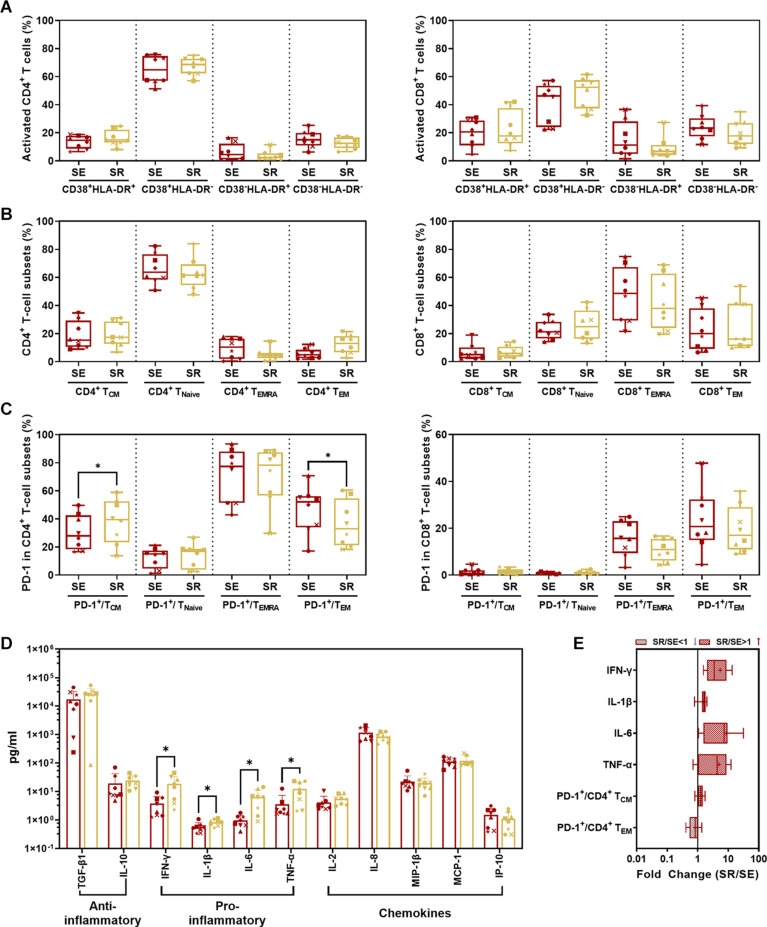
Comparison of CD4^+^/CD8^+^ T-cell subsets and cytokines between SR and SE phases. **(A)** Comparison of activated CD4^+^ (*left panel*) and CD8^+^ (*right panel*) T-cell subsets from PBMC in SR vs. SE. **(B)** Comparison of CD4^+^ (*left panel*) and CD8^+^ (*right panel*) memory T-cell subsets from PBMCs in SR vs. SE. The percentage of central memory CD4^+^/CD8^+^ T cells (CD4^+^/CD8^+^ T_CM_, CD3^+^ CD4^+^/CD8^+^ CCR7^+^ CD45RA^–^), naive CD4^+^/CD8^+^ T cells (CD4^+^/CD8^+^ T_Naive_, CD3^+^ CD4^+^/CD8^+^ CCR7^+^ CD45RA^+^), TEMRA cells (CD4^+^/CD8^+^ T_EMRA_, CD3^+^ CD4^+^/CD8^+^ CCR7^–^ CD45RA^+^), and effective memory T cells (CD4^+^/CD8^+^ T_EM_, CD3^+^ CD4^+^/CD8^+^ CCR7^–^ CD45RA^–^) were compared between SR and SE phases (paired *t*-test, *P* > 0.05). **(C)** Comparison of percentages of PD-1^+^ cells in memory T-cell subsets in SR vs. SE. Left panel, comparison of percentages of PD-1^+^ cells in CD4^+^ T_CM_, T_Naive_, T_EMRA_, and T_EM_ cells; right panel, comparison of percentages of PD-1^+^ cells in CD8^+^ T_CM_, T_Naive_, T_EMRA_, and T_EM_ cells. **(D)** Comparison of anti- or pro-inflammatory cytokines and chemokines between SR and SE phases. **(E)** The fold change of cytokines and T-cell subsets with the significant differences (SR value/SE value; red-filled patterns are the increased values, and gray-filled patterns are the decreased values). Data are mean ± SD from three independent experiments (^∗^*P* < 0.05, paired *t*-test, SR vs. SE).

Comparing SR to SE, we found no significant difference in activated CD4^+^/CD8^+^ T-cell subsets with CD38 and/or HLA-DR expression (Figure 7A) as well as memory T-cell subsets with CCR7 and/or CD45RA expression (Figure 7B) (*P* > 0.05). The only significant changes were an increase in PD-1^+^ CD4^+^ T_CM_ cells (*p* = 0.0110) and decrease in PD-1^+^ CD4^+^ T_EM_ cells (*P* = 0.0466) in SR compared to SE (Figure 7C, *left panel* and Figure 7E). IFN-γ (*P* = 0.0234), IL-1β (*P* = 0.0169), IL-6 (*P* = 0.0164), and TNF-α (*P* = 0.0435) were significantly elevated in SR compared to SE (Figures 7D,E).

### Systematic Comparison of the Level of Inflammation in ART-Treated SIVmac239-Infected Rhesus Macaques

**FIGURE 8:**
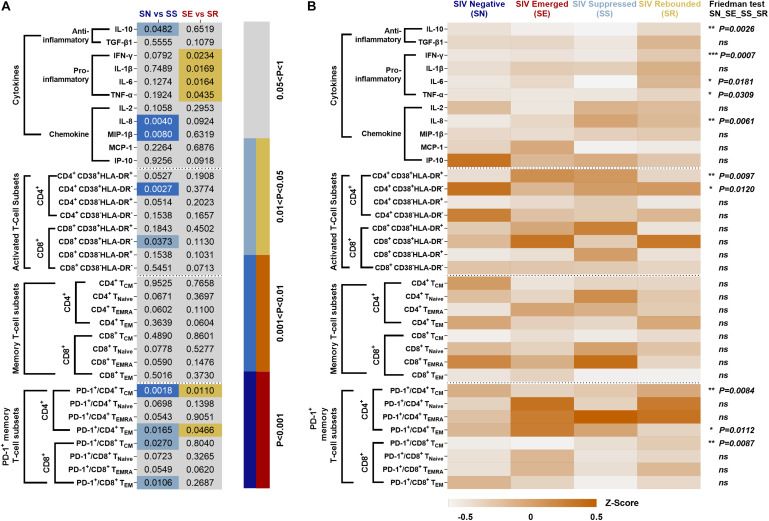
Systematic comparison of immune features in ART-treated, SIVmac239-infectecd macaques. **(A)** *P* values of immunocyte subsets and cytokines in SS vs. SN and SR vs. SE. **(B)** Expression pattern of immunocyte subsets and cytokines during SN, SE, SS, and SR stages. All data are standardized by z-score and analyzed by Friedman matched paired test (*P < 0.05; **P < 0.01; ***P < 0.001; ns, not significant).

Percentages of specific immune cells and cytokine levels in the four phases of “baseline-infection-treatment-withdrawal” were systematically compared in SN vs. SS and SE vs. SR (Figure 8A). The frequency of immunocyte types and cytokine expression levels were compared among the four stages (Figure 8B). Activated CD4^+^ T-cell subsets with high expression of CD38 or HLA-DR and memory CD4^+^/CD8^+^ T-cell subsets with high expression of PD-1 were consistently sustained from stage to stage.

## Discussion

Our comparison was based on longitudinal within-individual analyses of host–virus interaction on CD4^+^ T cells (supposed target) and CD8^+^ T cells (supposed effector). We systematically measured the subset of activation status in conjunction with their exhaustion status and cytokine profiling in non-human primate models. Our stage-dependent host–virus immunity comparison longitudinally tracked eight SIVmac239-infected rhesus macaques. We stratified the SIV persistent scenario by viral load representative of viral stimulation (acute) or persistence (chronic/latent) to obtain four individual-based stages. We compared SS to SN to reveal the host–virus interaction in the context of virus-undetectable host immunity status. Also, we compared SR to SE to reveal the host–virus interaction in the context of virus-detectable host immunity status. In each single stage, our findings were highly consistent with previous studies (Hunt et al., 2008; Molina-Pinelo et al., 2009; Robbins et al., 2009; Hunt, 2012; Vandergeeten et al., 2012; Wittkop et al., 2013; Osuji et al., 2018; Yero et al., 2019; Antar et al., 2020; Bordoni et al., 2020).

Comparing SS and SN, we found significantly elevated CD38^+^ activated CD4^+^/CD8^+^ T-cell subsets and PD-1^+^ memory CD4^+^/CD8^+^ T-cell subsets. T-cell activation by elevated CD38 and HLA-DR co-expression during HIV acute infection is commonly seen (Hua et al., 2014; Ndhlovu et al., 2015; Chen et al., 2017). It was worthy to note that CD38 expression indicated the activation against HIV and displays a tendency to exhaustion. HLA-DR was observed at the earlier phase of the acute infection and waned during the long-term SS stage. Therefore, the difference between SN and SS presented a CD38^+^HLA-DR^–^ subset. Furthermore, overexpressed PD-1 was shown between two stages. It has been documented that high expression of PD-1 and CD38 on CD8^+^ T cells is correlated with viral replication and disease progression in long-term chronic cases, indicating exhaustion of activated T cells (Hoffmann et al., 2016). Also, memory CD4^+^ T cells are continuously exhausted with overexpression of PD-1 (Evans et al., 2018). Collectively, our between-stage (SS vs. SN) comparison validated a dynamic process of activation/exhaustion during chronic latency along individual scenarios. Notably, such activation/exhaustion might produce “paradoxical” cytokines. Our comparison revealed MIP-1β, IL-8, and IL-10 as the most prominent. As ART suppresses dominant cytokines cascade into low levels (Hansen et al., 2009; Casetti et al., 2017; Sereti et al., 2017; Bordoni et al., 2020), pro-inflammatory MIP-1β and IL-8 and anti-inflammatory factor IL-10 are secreted to exaggerate the imbalance of host immunity. Also, such imbalance of cytokine microenvironment is likely to promote CD8^+^ T cells’ susceptibility to senescence/apoptosis, forming a chronic vicious circle. Therefore, our stage-dependent comparison validated the “paradoxical” activation/exhaustion of HIV/SIV-specific memory/effector T cells, which responded to the latent and continuous HIV/SIV antigen representation.

Comparing SR and SE, we found increased PD-1^+^CD4^+^ T_CM_ cells with decreased PD-1^+^CD4^+^ T_E__M_ cells and four remarkedly increased pro-inflammatory cytokines. These results suggested that the targeted CD4^+^ T cells were undergoing exhaustion and senescence. During HIV replication, CD4^+^ T cells were impaired, resulting in reduced immunity hemostasis. In previous studies, the majority of PD-1 is overexpressed on the memory CD8^+^ T cells in both SR and SE stages (Hansen et al., 2009), which could explain the low disparity between the two stages. Regarding the cytokines, non-specific and robust pro-inflammatory cytokines including TNF-α, IL-6, and IFN-γ (Regidor et al., 2011; Vandergeeten et al., 2012; Osuji et al., 2018) were significantly elevated. Such pro-inflammatory profiling indicated that the host immunity was hyperactive and imbalanced. It was paradoxical that such robust cytokines could not eliminate the rebounding virus, which was also attributed to the imbalanced cytokine shift. As the SR stage occurred after a long host–virus interaction undergone ART treatment, it meant that host global immunocompetence was eventually shifted from activation/exhaustion to exhaustion/senescence, reflected by our differential cytokine profiling between the two stages (SE vs. SR).

Our study had numerous limitations. First, the size of our non-primate monkeys was relatively small, leading to the low validity of the present findings. Secondly, we merely measured PD-1 among a bundle of checkpoint receptors, such as Tim-3 and Lag-3, to represent immune checkpoint receptors on CD4 and CD8^+^ T cells. Thirdly, the profound polyfunctionality of CD4^+^ and CD8^+^ T cells and cytokine profiles have not been comprehensively measured. Fourthly, the dynamic trajectory on CD4^+^ and CD8^+^ T-cell functionality and cytokine profiles was not individually tracked and explained by intra-individual and inter-individual variation due to the small size and low frequency of measurements. Fifthly, the co-expression was not joint analyzed as most combinations of multiple phenotypes were functionally undetermined.

In summary, our stage-dependent individual-traced comparison has presented an eventual shifting tendency from activation/exhaustion to exhaustion/senescence using eight rhesus macaques undergoing four stages. It was supposed that sustained expression of PD-1 attempted to check the point and maintain the balance, yet actually accelerated the immune activation and exhausted the immunity response. The dynamic imbalance and shift of host immunity during the chronic scenario could be individually seen using the present within-individual comparison. Overall, this stage-dependent longitudinal comparison further confirmed that SIV accelerates host immunosenescence continuously independent of viral replication, the findings of which may provide insights into identifying targets for novel intervention reducing persistent immunoactivation in ART-treated individuals.

## Data Availability Statement

The original contributions presented in the study are included in the article/supplementary material, further inquiries can be directed to the corresponding authors.

## Ethics Statement

The animal study was reviewed and approved by Institutional Animal Care and Use Committee (IACUC) of the Institute of Laboratory Animal Science, Chinese Academy of Medical Sciences.

## Author Contributions

JX: conceptualization and writing—review and editing. ZC and LTo: methodology. LTo, ZC, LTi, JZ, JL, QL, and TC: investigation. LTo and YW: statistical analysis. LTo and JX: writing—original draft. JX, QW, and ZC: funding acquisition. JX and QW: resources and supervision. All authors contributed to the article and approved the submitted version.

## Conflict of Interest

The authors declare that the research was conducted in the absence of any commercial or financial relationships that could be construed as a potential conflict of interest.

## Footnotes

1 http://www.acmedsci.ac.uk/more/news/the-use-of-non-human-primates-in-research/

## References

[B1] Antar A. A. Jenike K. M. Jang S. Rigau D. N. Reeves D. B. Hoh R. (2020). Longitudinal study reveals HIV-1-infected CD4+ T cell dynamics during long-term antiretroviral therapy. *J. Clin. Invest.* 130 3543–3559. 10.1172/jci135953 32191639 PMC7324206

[B2] Appay V. Almeida J. R. Sauce D. Autran B. Papagno L. (2007). Accelerated immune senescence and HIV-1 infection. *Exp. Gerontol.* 42 432–437. 10.1016/j.exger.2006.12.003 17307327

[B3] Appay V. Sauce D. (2017). Assessing immune aging in HIV-infected patients. *Virulence* 8 529–538. 10.1080/21505594.2016.1195536 27310730 PMC5538339

[B4] Booiman T. Wit F. W. Girigorie A. F. Maurer I. De Francesco D. Sabin C. A. (2017). Terminal differentiation of T cells is strongly associated with CMV infection and increased in HIV-positive individuals on ART and lifestyle matched controls. *PLoS One* 12:e0183357. 10.1371/journal.pone.0183357 28806406 PMC5555623

[B5] Bordoni V. Sacchi A. Casetti R. Cimini E. Tartaglia E. Pinnetti C. (2020). Impact of ART on dynamics of growth factors and cytokines in primary HIV infection. *Cytokine* 125:154839. 10.1016/j.cyto.2019.154839 31542514

[B6] Brenchley J. M. Price D. A. Schacker T. W. Asher T. E. Silvestri G. Rao S. (2006). Microbial translocation is a cause of systemic immune activation in chronic HIV infection. *Nat. Med.* 12 1365–1371. 17115046 10.1038/nm1511

[B7] Cao W. Mehraj V. Kaufmann D. E. Li T. Routy J. P. (2016). Elevation and persistence of CD8 T-cells in HIV infection: the Achilles heel in the ART era. *J. Int. AIDS Soc.* 19:20697. 10.7448/ias.19.1.20697 26945343 PMC4779330

[B8] Casetti R. Pinnetti C. Sacchi A. De Simone G. Bordoni V. Cimini E. (2017). HIV-specific CD8 T cells producing CCL-4 are associated with worse immune reconstitution during chronic infection. *J. Acquir. Immune Defic. Syndr.* 75 338–344. 10.1097/qai.0000000000001392 28418988

[B9] Chen P. Su B. Zhang T. Zhu X. Xia W. Fu Y. (2017). Perturbations of monocyte subsets and their association with T helper cell differentiation in acute and chronic HIV-1-infected patients. *Front. Immunol.* 8:272. 10.3389/fimmu.2017.00272 28348563 PMC5347116

[B10] Chong H. Xue J. Zhu Y. Cong Z. Chen T. Wei Q. (2019). Monotherapy with a low-dose lipopeptide HIV fusion inhibitor maintains long-term viral suppression in rhesus macaques. *PLoS Pathog.* 15:e1007552. 10.1371/journal.ppat.1007552 30716118 PMC6375636

[B11] Day C. L. Kaufmann D. E. Kiepiela P. Brown J. A. Moodley E. S. Reddy S. (2006). PD-1 expression on HIV-specific T cells is associated with T-cell exhaustion and disease progression. *Nature* 443 350–354. 16921384 10.1038/nature05115

[B12] Deeks S. G. (2011). HIV infection, inflammation, immunosenescence, and aging. *Annu. Rev. Med.* 62 141–155. 10.1146/annurev-med-042909-093756 21090961 PMC3759035

[B13] Deeks S. G. Lewin S. R. Havlir D. V. (2013). The end of AIDS: HIV infection as a chronic disease. *Lancet* 382 1525–1533. 10.1016/s0140-6736(13)61809-7 24152939 PMC4058441

[B14] Dock J. N. Effros R. B. (2011). Role of CD8 T cell replicative senescence in human aging and in HIV-mediated immunosenescence. *Aging Dis.* 2 382–397. 22308228 PMC3269814

[B15] Douek D. C. (2013). Immune activation, HIV persistence, and the cure. *Top. Antivir. Med.* 21 128–132. 24225078 PMC6148844

[B16] Evans V. A. van der Sluis R. M. Solomon A. Dantanarayana A. Mcneil C. Garsia R. (2018). Programmed cell death-1 contributes to the establishment and maintenance of HIV-1 latency. *AIDS* 32 1491–1497. 10.1097/qad.0000000000001849 29746296 PMC6026054

[B17] Goepfert P. A. Bansal A. Edwards B. H. Ritter G. D. Jr. Tellez I. Mcpherson S. A. (2000). A significant number of human immunodeficiency virus epitope-specific cytotoxic T lymphocytes detected by tetramer binding do not produce gamma interferon. *J. Virol.* 74 10249–10255. 10.1128/jvi.74.21.10249-10255.2000 11024158 PMC102068

[B18] Goulder P. J. Tang Y. Brander C. Betts M. R. Altfeld M. Annamalai K. (2000). Functionally inert HIV-specific cytotoxic T lymphocytes do not play a major role in chronically infected adults and children. *J. Exp. Med.* 192 1819–1832. 10.1084/jem.192.12.1819 11120778 PMC2213508

[B19] Hansen S. G. Vieville C. Whizin N. Coyne-Johnson L. Siess D. C. Drummond D. D. (2009). Effector memory T cell responses are associated with protection of rhesus monkeys from mucosal simian immunodeficiency virus challenge. *Nat. Med.* 15 293–299. 10.1038/nm.1935 19219024 PMC2720091

[B20] Hansson G. K. (2005). Inflammation, atherosclerosis, and coronary artery disease. *N. Engl. J. Med.* 352 1685–1695. 10.1056/nejmra043430 15843671

[B21] Hoffmann M. Pantazis N. Martin G. E. Hickling S. Hurst J. Meyerowitz J. (2016). Exhaustion of activated CD8 T cells predicts disease progression in primary HIV-1 infection. *PLoS Pathog.* 12:e1005661. 10.1371/journal.ppat.1005661 27415828 PMC4945085

[B22] Hua S. Lécuroux C. Sáez-Cirión A. Pancino G. Girault I. Versmisse P. (2014). Potential role for HIV-specific CD38-/HLA-DR+ CD8+ T cells in viral suppression and cytotoxicity in HIV controllers. *PLoS One* 9:e101920. 10.1371/journal.pone.0101920 25000587 PMC4084978

[B23] Hunt P. W. (2012). HIV and inflammation: mechanisms and consequences. *Curr. HIV/AIDS Rep.* 9 139–147. 10.1007/s11904-012-0118-8 22528766

[B24] Hunt P. W. Brenchley J. Sinclair E. Mccune J. M. Roland M. Page-Shafer K. (2008). Relationship between T cell activation and CD4+ T cell count in HIV-seropositive individuals with undetectable plasma HIV RNA levels in the absence of therapy. *J. Infect. Dis.* 197 126–133. 10.1086/524143 18171295 PMC3466592

[B25] Kaufmann D. E. Walker B. D. (2008). Programmed death-1 as a factor in immune exhaustion and activation in HIV infection. *Curr. Opin. HIV AIDS* 3 362–367. 10.1097/coh.0b013e3282f9ae8b 19372991

[B26] Kostense S. Ogg G. S. Manting E. H. Gillespie G. Joling J. Vandenberghe K. (2001). High viral burden in the presence of major HIV-specific CD8(+) T cell expansions: evidence for impaired CTL effector function. *Eur. J. Immunol.* 31 677–686. 10.1002/1521-4141(200103)31:3<677::aid-immu677>3.0.co;2-m 11241270

[B27] Kostense S. Vandenberghe K. Joling J. Van Baarle D. Nanlohy N. Manting E. (2002). Persistent numbers of tetramer+ CD8(+) T cells, but loss of interferon-gamma+ HIV-specific T cells during progression to AIDS. *Blood* 99 2505–2511. 10.1182/blood.v99.7.2505 11895786

[B28] Lichterfeld M. Yu X. G. Mui S. K. Williams K. L. Trocha A. Brockman M. A. (2007). Selective depletion of high-avidity human immunodeficiency virus type 1 (HIV-1)-specific CD8+ T cells after early HIV-1 infection. *J. Virol.* 81 4199–4214. 10.1128/jvi.01388-06 17287271 PMC1866095

[B29] Martínez-Zamudio R. I. Dewald H. K. Vasilopoulos T. Gittens-Williams L. Fitzgerald-Bocarsly P. Herbig U. (2021). Senescence-associated β-galactosidase reveals the abundance of senescent CD8+ T cells in aging humans. *Aging Cell* 20:e13344. 33939265 10.1111/acel.13344 PMC8135084

[B30] Molina-Pinelo S. Vallejo A. Díaz L. Soriano-Sarabia N. Ferrando-Martínez S. Resino S. (2009). Premature immunosenescence in HIV-infected patients on highly active antiretroviral therapy with low-level CD4 T cell repopulation. *J. Antimicrob. Chemother.* 64 579–588. 10.1093/jac/dkp248 19608579

[B31] Monti D. Ostan R. Borelli V. Castellani G. Franceschi C. (2017). Inflammaging and human longevity in the omics era. *Mech. Ageing Dev.* 165 129–138. 10.1016/j.mad.2016.12.008 28038993

[B32] Muenchhoff M. Adland E. Roider J. Kløverpris H. Leslie A. Boehm S. (2019). Differential pathogen-specific immune reconstitution in antiretroviral therapy-treated human immunodeficiency virus-infected children. *J. Infect. Dis.* 219 1407–1417. 10.1093/infdis/jiy668 30624717 PMC6467189

[B33] Naeger D. M. Martin J. N. Sinclair E. Hunt P. W. Bangsberg D. R. Hecht F. (2010). Cytomegalovirus-specific T cells persist at very high levels during long-term antiretroviral treatment of HIV disease. *PLoS One* 5:e8886. 10.1371/journal.pone.0008886 20126452 PMC2813282

[B34] Ndhlovu Z. M. Kamya P. Mewalal N. Kløverpris H. N. Nkosi T. Pretorius K. (2015). Magnitude and kinetics of CD8+ T cell activation during hyperacute HIV infection impact viral set point. *Immunity* 43 591–604. 10.1016/j.immuni.2015.08.012 26362266 PMC4575777

[B35] Osuji F. N. Onyenekwe C. C. Ahaneku J. E. Ukibe N. R. (2018). The effects of highly active antiretroviral therapy on the serum levels of pro-inflammatory and anti-inflammatory cytokines in HIV infected subjects. *J. Biomed. Sci.* 25:88. 30501642 10.1186/s12929-018-0490-9 PMC6276218

[B36] Paiardini M. Müller-Trutwin M. (2013). HIV-associated chronic immune activation. *Immunol. Rev.* 254 78–101. 10.1111/imr.12079 23772616 PMC3729961

[B37] Pathai S. Bajillan H. Landay A. L. High K. P. (2014). Is HIV a model of accelerated or accentuated aging? *J. Gerontol. A Biol. Sci. Med. Sci.* 69 833–842. 10.1093/gerona/glt168 24158766 PMC4067117

[B38] Petrovas C. Chaon B. Ambrozak D. R. Price D. A. Melenhorst J. J. Hill B. J. (2009). Differential association of programmed death-1 and CD57 with ex vivo survival of CD8+ T cells in HIV infection. *J. Immunol.* 183 1120–1132. 10.4049/jimmunol.0900182 19564339 PMC2923541

[B39] Regidor D. L. Detels R. Breen E. C. Widney D. P. Jacobson L. P. Palella F. (2011). Effect of highly active antiretroviral therapy on biomarkers of B-lymphocyte activation and inflammation. *AIDS* 25 303–314. 10.1097/qad.0b013e32834273ad 21192231 PMC3322644

[B40] Robbins G. K. Spritzler J. G. Chan E. S. Asmuth D. M. Gandhi R. T. Rodriguez B. A. (2009). Incomplete reconstitution of T cell subsets on combination antiretroviral therapy in the AIDS Clinical Trials Group protocol 384. *Clin. Infect. Dis.* 48 350–361. 10.1086/595888 19123865 PMC2676920

[B41] Sáez-Cirión A. Sereti I. (2021). Immunometabolism and HIV-1 pathogenesis: food for thought. *Nat. Rev. Immunol.* 21 5–19. 10.1038/s41577-020-0381-7 32764670

[B42] Schouten J. Wit F. W. Stolte I. G. Kootstra N. A. Van Der Valk M. Geerlings S. E. (2014). Cross-sectional comparison of the prevalence of age-associated comorbidities and their risk factors between HIV-infected and uninfected individuals: the AGEhIV cohort study. *Clin. Infect. Dis.* 59 1787–1797. 25182245 10.1093/cid/ciu701

[B43] Sereti I. Krebs S. J. Phanuphak N. Fletcher J. L. Slike B. Pinyakorn S. (2017). Persistent, albeit reduced, chronic inflammation in persons starting antiretroviral therapy in acute HIV infection. *Clin. Infect. Dis.* 64 124–131. 10.1093/cid/ciw683 27737952 PMC5215214

[B44] Streeck H. Jolin J. S. Qi Y. Yassine-Diab B. Johnson R. C. Kwon D. S. (2009). Human immunodeficiency virus type 1-specific CD8+ T-cell responses during primary infection are major determinants of the viral set point and loss of CD4+ T cells. *J. Virol.* 83 7641–7648. 10.1128/jvi.00182-09 19458000 PMC2708622

[B45] van den Dries L. Claassen M. A. A. Groothuismink Z. M. A. van Gorp E. Boonstra A. (2017). Immune activation in prolonged cART-suppressed HIV patients is comparable to that of healthy controls. *Virology* 509 133–139. 10.1016/j.virol.2017.06.014 28644978

[B46] van der Most R. G. Murali-Krishna K. Lanier J. G. Wherry E. J. Puglielli M. T. Blattman J. N. (2003). Changing immunodominance patterns in antiviral CD8 T-cell responses after loss of epitope presentation or chronic antigenic stimulation. *Virology* 315 93–102. 10.1016/j.virol.2003.07.001 14592762

[B47] Vandergeeten C. Fromentin R. Chomont N. (2012). The role of cytokines in the establishment, persistence and eradication of the HIV reservoir. *Cytokine Growth Factor Rev.* 23 143–149. 10.1016/j.cytogfr.2012.05.001 22743037 PMC3767481

[B48] Whitney J. B. Lim S. Y. Osuna C. E. Kublin J. L. Chen E. Yoon G. (2018). Prevention of SIVmac251 reservoir seeding in rhesus monkeys by early antiretroviral therapy. *Nat. Commun.* 9:5429. 30575753 10.1038/s41467-018-07881-9 PMC6303321

[B49] Wittkop L. Bitard J. Lazaro E. Neau D. Bonnet F. Mercie P. (2013). Effect of cytomegalovirus-induced immune response, self antigen-induced immune response, and microbial translocation on chronic immune activation in successfully treated HIV type 1-infected patients: the ANRS CO3 Aquitaine Cohort. *J. Infect. Dis.* 207 622–627. 23204178 10.1093/infdis/jis732

[B50] Yero A. Farnos O. Rabezanahary H. Racine G. Estaquier J. Jenabian M. A. (2019). Differential dynamics of regulatory T-cell and Th17 cell balance in Mesenteric lymph nodes and blood following early antiretroviral initiation during acute simian immunodeficiency virus infection. *J. Virol.* 93:e00371–19. 31315987 10.1128/JVI.00371-19 PMC6744245

[B51] Youngblood B. Noto A. Porichis F. Akondy R. S. Ndhlovu Z. M. Austin J. W. (2013). Cutting edge: prolonged exposure to HIV reinforces a poised epigenetic program for PD-1 expression in virus-specific CD8 T cells. *J. Immunol.* 191 540–544. 10.4049/jimmunol.1203161 23772031 PMC3702641

[B52] Zhang J. Y. Zhang Z. Wang X. Fu J. L. Yao J. Jiao Y. (2007). PD-1 up-regulation is correlated with HIV-specific memory CD8+ T-cell exhaustion in typical progressors but not in long-term nonprogressors. *Blood* 109 4671–4678. 10.1182/blood-2006-09-044826 17272504

